# Seasonal influenza immunization in early infancy?

**DOI:** 10.1186/1471-2458-12-873

**Published:** 2012-10-15

**Authors:** Maurizio Bonati, Antonio Clavenna

**Affiliations:** 1 Laboratory for Mother and Child Health, Department of Public Health, Mario Negri Research Institute, Via G. La Masa 19, 20156, Milan, Italy; 2 Pharmacoepidemiology Unit, Mario Negri Research Institute of Milan, Milan, Italy

**Keywords:** Influenza vaccines, Vaccination, Newborn, Infant

## 

Seasonal influenza is an important public health and medical challenge. Vaccination against influenza is recognized worldwide as the main strategy for prevention and control. The findings of meta-analyses, however, suggest that efficacy and effectiveness of influenza vaccines, both in people aged 65 years or older [1] and in children, are lower than believed [2]. These findings were recently confirmed, even if mitigated (only) a little, by an additional meta-analysis. This recent study assessed efficacy and effectiveness of licensed influenza vaccines in the USA with sensitive and highly specific diagnostic tests used to confirm influenza [3]. Findings show that in healthy children influenza vaccines have a very variable efficacy (vaccine effects on lab-confirmed influenza) and effectiveness (prevention of influenza-like illness). Available safety data are scant, particularly for inactivated vaccines in younger children [2]. In such a context, when the seasonal periods for influenza arrive, questions concerning who should receive the influenza vaccine are raised among health operators and lay people worldwide. Pediatricians, parents, and caregivers of infants and children in particular, are doubtful as to whether to recommend or accept influenza immunization of children or not [4], also because in children the infection is usually self-limiting without complications, though the risk is higher in those aged less than 6 months [5].

In 2010, the Advisory Committee on Immunization Practices (ACIP) established the first recommendation for a national, universal seasonal influenza vaccination in the USA [6].

Vaccination every year is recommended by ACIP with trivalent inactivated vaccine (TIV) for all individuals aged 6 months or older, or with live attenuated influenza vaccine (LAIV) for healthy, non-pregnant people aged 2-49 years. Up until now only a few countries (i.e., Austria, Canada, and Estonia) universally recommend the seasonal influenza vaccination. The majority of countries continues to apply, for the paediatric population, the World Health Organization recommendations that suggest vaccinating only children older than 6 months of age with certain coexisting conditions (chronic heart or lung diseases, metabolic or renal diseases, chronic neurological conditions, or immunodeficiencies) through strategies that involve an active promotion of the vaccine [7]. Household contacts of those children are unfortunately not considered, although individual member states may consider influenza vaccination programs that target all persons 6 months of age and older if feasible and cost-effective.

This is the public health context, in which national and regional decisions are the result of prevailing evaluations often characterized by a balancing of, and mediation between, evidence based knowledge, available public funds, health system organization, as well as historical legacies and inertia, policy lock-in, and unchecked assumptions about the benefit of influenza vaccines.

Scant data are available concerning the incidence of influenza in the pediatric population. A meta-analysis of 24 epidemiological studies monitoring different influenza seasons estimated an annual incidence in children less than 5 years old of 5.6% (95%CI 2.8 to 10.6%) in the developed countries and of 15.0% (95%CI 9.8 to 22.9%) in developing countries [5]. The incidence in developed countries decreased to 3.0% (0.9, 10.0%) when only study using polymerase chain reaction as diagnostic tool were taken into account [5].

The average annual rate of hospitalization associated with influenza is reported in developed countries as ranging from 0.6 to 2.7 per 1000 children younger than 5 years of age [8,9], whereas the average annual rates of outpatient visits attributable to influenza are 10-250 times as high as hospitalization rates, and increase with age [8].

This wide variability in estimates is attributable to differences in the studies performed. These include study year, country, setting, size and characteristics of population surveyed, definition of influenza season, study design and objectives.

Such a weakly defined epidemiological profile coincides with the lack of hard evidence on efficacy and effectiveness of influenza immunization in children because the same limitations are present in clinical trials, with even further variability attributable to laboratory tests, vaccines used, etc.

Public health decisions and recommendations are consequently not only complex in and of themselves, but are also difficult in such a scenario.

Ongoing research in the field (much of which is industry sponsored) is waiting for a new generation of more effective, cross-protective influenza vaccines and is, in the meantime, oriented towards defining the efficacy in children of influenza vaccines that are used in adults. The TIV vaccine has currently been shown an efficacy (prevention of confirmed influenza) of 59% and an effectiveness (prevention of influenza-like illness) of 36% in children older than 2 years of age [2], whereas the intranasal LAIV has been shown to have a greater efficacy (69.2-95.6%) only in children 2-7 years of age, but cannot be used in children under 2 years of age because of an increased risk of hospitalization [10].

At present, children aged 6-72 months are the favorite population of influenza vaccine studies to show a superior protective efficacy of adjuvant TIV vaccines [11].

The question, however, remains. Should infants be vaccinated against seasonal influenza? Currently, this vaccination is not recommended in any country for healthy, preterm, or low birth weight infants, even if they have chronic diseases that are contemplated by immunization strategies for infants older than 6 months. Moreover, it is important to underline that influenza vaccines up to date have not been approved by regulators for use in infants.

Due to the immature or impaired cellular and humoral immune systems and the presence of maternal antibodies at protective levels in babies, in several countries vaccines are routinely recommended from 2 months of age, also for preterm and low birth weight infants [12,13]. The only exception is the vaccination against hepatitis B for all at risk newborns, in particular those born to mothers HBsAG positive, who should receive the vaccine within 12-24 hours after birth, regardless of gestational age or birth weight [12,14].

Although the risk of severe influenza infection and the rate of hospitalization are higher in infants during their first 6 months of life than in older infants [8], in particular in those born to mothers who have not been exposed to the virus [15], there are only 4 published studies on inactivated influenza vaccination in infants under 6 months [16-19].

In the first study, 62 infants 3 to 5 months of age with bronchopulmonary dysplasia or congenital heart disease were vaccinated using four different TIV vaccines [16]. In the second study, 42 healthy infants aged 10-22 weeks received two different TIV vaccines [17]. In the third, 126 infants 2-3 months of age were randomized to receive a TIV vaccine either via the intramuscular or intradermal route [18]. The findings of the first two studies demonstrated seroconversion only in the range of 0-55% against the various vaccine-contained antigens with a suggested age related trend, whereas the extremely high percentage of maternal antibodies at protective levels in the infants in the third study precluded the testing of immunogenicity in this age group (only 4 out of 126 infants had hemagglutination inhibition titer <40 against at least one vaccine-covered antigen before vaccination) [18].

In a large randomized placebo controlled trial evaluating the antibody titers in 1304 subjects 6-12 weeks of age, 90.2% of TIV recipients achieved potential seroprotection to at least one influenza strain following the second vaccine dose [19]. Seroprotection rates differed from 10.9 to 85.6% among individual vaccine antigens. Safety profiles were similar in the TIV and placebo groups [19].

All these studies were mainly focused on the evaluation of immunogenicity. None of them was designed for evaluating clinical or public health endpoints.

In the past a few studies were performed to evaluate the immunogenicity of LAIV vaccines in infants under 6 months [2,20,21]. The rate of seroconversion was similar to that observed with TIV vaccines, but a higher rate of adverse effects was reported with LAIV vaccines in children under 2 years of age [10].

Without evidence suggesting benefit outweights harms at either the individual or societal (public health) level, today, any attempt to suggest immunizing infants less than 6 months of age against seasonal influenza would be off-label, arbitrary and unethical. Off-label use of drugs and vaccines is legal since regulatory agencies do not regulate the practice of medicine but, either way, it should be evidence-based. Public health solutions using current influenza vaccines are difficult to imagine because of the wide methodological differences between the performed studies; the interseasonal variation and the everchanging antigenicity of the influenza virus; the variation in efficacy and effectiveness (risk reduction in populations offered the vaccine) by total vaccine coverage and degree of herd immunity for each season.

However, infants younger than 6 months are also a reservoir for potential child-to-adult transmission and other effective preventive and treatment strategies for these infants alternative to influenza vaccines should be fully evaluated. The high maternal antibody levels detected in many infants in the above studies are in agreement with the results of investigations showing that the vaccine is safe and effective in protecting pregnant women and their infants for a few months after birth [22]. 45-65% of influenza disease in infants is preventable through maternal immunization programmes [23]. In 2005, the WHO was already recommending influenza vaccination for all pregnant women during the influenza season [24]. Although new evidences supports the efficacy and the effectiveness of influenza vaccine administration during pregnancy in exposed women and their newborn babies [25,26], and safety data suggest inactivated vaccines for seasonal influenza are safe in pregnancy [27,28], vaccination is underused in pregnancy [29].

Concerning the passive immunization, or indirect protection, of a newborn, the breast milk-mediated protection against respiratory viruses is well established [30], as the role of brestfeeding in infants infected with influenza virus [31], thus promoting exclusive breastfeeding also against infectious diseases should be kept in mind [32,33]. Therefore, while waiting for safe, effective and affordable vaccines for infants, this population can also be protected by limiting their exposure to influenza through both educational interventions (including hand washing) [6,34,35] targeting health care workers [36], family members, caregivers, and all individuals who reside with newborns, in particular ill ones [35]. Moreover, some benefits may be obtained by applying “cocoon” immunization strategies immunizing all individuals who come into contact with infants, as suggested by uncontrolled studies [37].

In conclusion, seasonal influenza is a major public health issue at the extremes of ages and in susceptible groups. Until an effective influenza vaccine is achievable, more evidence about both efficacy of flu immunization in early infants and alternative interventions to influenza vaccination is available, and preventive strategies implemented, it would be more important to improve the coverage in higher risk children than to enlarge the immunization age range of healthy children.

## Abbreviations

ACIP: Advisory Committee on Immunization Practices; LAIV: Live attenuated influenza vaccine; TIV: Trivalent inactivated vaccine.

## Competing interest

Both authors declare that about competing interest have nothing to declare.

## Authors’ contributions

MB, Head of Public Health Department and of the Laboratory of Mother and Child Health at Mario Negri Research Institute of Milan, was responsible for the drafting, revising, and submitting of the commentary; AC, Head of Pharmacoepidemiology Unit at Mario Negri Research Institute of Milan reviewed and revised the text for important intellectual content. Both authors read and approved the final manuscript.

## Pre-publication history

The pre-publication history for this paper can be accessed here:

http://www.biomedcentral.com/1471-2458/12/873/prepub
